# Advanced tumor electric fields therapy: A review of innovative research and development and prospect of application in glioblastoma

**DOI:** 10.1111/cns.14720

**Published:** 2024-05-07

**Authors:** Jinxin Lan, Yuyang Liu, Junyi Chen, Hongyu Liu, Yaping Feng, Jialin Liu, Ling Chen

**Affiliations:** ^1^ Department of Neurosurgery Chinese PLA General Hospital Beijing China; ^2^ School of Medicine Nankai University Tianjin China; ^3^ Medical School of Chinese PLA Beijing China; ^4^ Department of Neurosurgery 920th Hospital of Joint Logistics Support Force Kunming China; ^5^ Department of Neurosurgery Hainan Hospital of Chinese PLA General Hospital Hainan China

**Keywords:** combination therapy, glioblastoma multiforme, physical therapy, tumor electric fields therapy

## Abstract

**Background:**

Glioblastoma multiforme (GBM) is an aggressive malignant tumor with a high mortality rate and is the most prevalent primary intracranial tumor that remains incurable. The current standard treatment, which involves surgery along with concurrent radiotherapy and chemotherapy, only yields a survival time of 14–16 months. However, the introduction of tumor electric fields therapy (TEFT) has provided a glimmer of hope for patients with newly diagnosed and recurrent GBM, as it has been shown to extend the median survival time to 20 months. The combination of TEFT and other advanced therapies is a promising trend in the field of GBM, facilitated by advancements in medical technology.

**Aims:**

In this review, we provide a concise overview of the mechanism and efficacy of TEFT. In addition, we mainly discussed the innovation of TEFT and our proposed blueprint for TEFT implementation.

**Conclusion:**

Tumor electric fields therapy is an effective and highly promising treatment modality for GBM. The full therapeutic potential of TEFT can be exploited by combined with other innovative technologies and treatments.

## Graphical abstract

The prospect of TEFT plus advanced therapy including PDT, ultrasound device, universal implantable electrodes, NovoTAL System and digital twins (By Figdraw). In combination with a variety of physiotherapy innovations, we envision a multimodal treatment approach that improves TEFT antitumor efficacy, while providing an excellent safety profile.

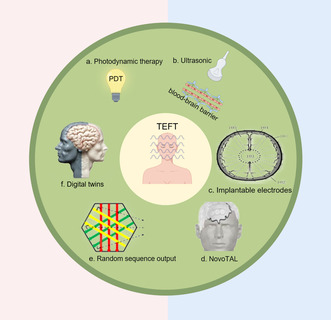

## 1 INTRODUCTION

Glioblastoma multiforme (GBM) is a highly aggressive and prevalent malignant primary brain tumor, accounting for 6.9% of 5‐year survival rates and 14.2% of all tumors, as well as 50.1% of all malignant tumors.^1^ Unfortunately, the prognosis for GBM is consistently poor, and there are limited successful therapies available in clinical practice. Despite undergoing standard treatment, which involves maximal safe surgical resection followed by concomitant radiotherapy and chemotherapy, GBM remains incurable, with a median overall survival of 14.6 months.^2^ Both temozolomide (TMZ) and radiotherapy are known to elicit symptoms of nausea and vomiting.^3^ Additionally, chemotherapy induces various adverse effects, including myelosuppression, alopecia, and fatigue, among others. The clinical utility of chemotherapy is constrained by its limited efficacy in the face of drug resistance, particularly in the case of TMZ.^4^ TMZ resistance is a significant factor contributing to a negative prognosis in patients.^4^ Radiotherapy has been associated with the development of radiation‐induced brain injury, which includes cognitive dysfunction and increased intracranial pressure.^5^, ^6^ In cases of recurrent GBM, the median overall survival (OS) is typically limited to 3–5 months without the implementation of effective therapeutic interventions.^7^ Consequently, the investigation of an anticancer treatment modality that is both efficacious and well tolerated is crucial for enhancing the survival rates of individuals diagnosed with GBM.

Tumor electric fields therapy (TEFT) is a biophysical technology that inhibits the growth of proliferating cells, including cancer cells, while sparing nonproliferating cells when applied under appropriate conditions.^8^ Dr.Ling Chen's team, along with other researchers, has demonstrated that TEFT, which utilizes alternating electric fields of low intensity (1–3 V/cm) and intermediate frequency (100–300 kHz), induces cell death in a wide range of tumor cells both in vitro and in vivo^9^, ^10^, ^11^ while having minimal impact on normal cells.^8^ It has been observed that most types of GBM cells exhibit optimal response at a frequency of 200 kHz, with only a few cell types showing no response at this frequency.^11^ The effectiveness of TEFT on cellular processes such as cell division and cell death was found to be influenced by the intensity of the applied electric field and the angle between the electric field and the axis of division.^9^ As a form of physical treatment therapy, TEFT demonstrated a favorable safety profile with minimal adverse reactions, primarily limited to skin‐related adverse events.^9^, ^12^, ^13^ A clinical trial involving 10 patients with recurrent GBM treated with TEFT revealed a median OS of 62.2 weeks. Based on these findings, TEFT received approval in the United States and Europe for the treatment of recurrent GBM and is now recommended as a first‐line therapy following surgical resection, radiotherapy, and TMZ. A more extensive clinical trial of EF‐14, which encompassed 695 patients, revealed that the median OS was 20.9 months in the TEFT plus TMZ group, whereas it was 16.0 months in the TMZ alone group. Additionally, the median progression‐free survival (PFS) was observed to be 6.7 months in the TEFT plus TMZ group, compared to 4.0 months in the TMZ group.^14^ Notably, for Chinese patients, the median PFS was 16 months in the TEFT plus TMZ group, in contrast to 11 months in the TMZ group. Similarly, the median OS for Chinese patients was 21.8 months in the TEFT plus TMZ group, while it was 15 months in the TMZ group.^15^ It is worth mentioning that the TEFT instruments were granted approval by the Chinese National Medical Products Administration in 2020.

Despite the significant improvement in the prognosis of patients with GBM through the use of TEFT, there remains a limited understanding of the molecular mechanisms underlying TEFT action. Furthermore, it is crucial to optimize and upgrade the current hardware and software to enhance the efficacy of TEFT. Consequently, this article aims to summarize the recently proposed mechanisms by which TEFT induces antitumor effects and to discuss the prospects of optimizing TEFT instruments.

## 2 MECHANISM OF TEFT

The mechanisms underlying tumor cytotoxicity can be categorized into several perspectives, including apoptosis, autophagy, cell cycle arrest, anti‐angiogenesis, enhanced drug penetration, reduced DNA repair capacity, diminished migration and invasion capabilities, and immune activation.^13^, ^16^ Preclinical studies have shown multiple effects on GBM cells, including promoting cell death, inhibiting DNA repair, inhibiting proliferation, and regulating immune response. TEFT treatment results in the extension of mitosis in the majority of treated GBM cells, resulting in the cessation of proliferation. Additionally, approximately 25% of GBM cells undergoing mitosis experience destruction due to cell membrane rupture, while nuclear rotation is observed in a subset of cells.^8^

### 2.1 TEFT promoting several types of cell death

The primary mechanism employed in the application of TEFT involved anti‐mitotic effects, such as the induction of prolonged mitosis, aberrant mitotic morphology, and mitotic cell death.^16^, ^17^ The principal function of the mitotic spindle is to accurately segregate the chromosomes to opposing poles of the cells.^18^ TEFT has been found to impair chromosomal segregation and cell division through two major mechanisms, as summarized in several reviews. These mechanisms include the disruption of mitotic spindle microtubule formation and the dielectrophoretic effect.^19^, ^20^ Specifically, TEFT hinders the localization of cytokinetic cleavage furrow to the midline of the spindle by affecting Septin, resulting in plasma membrane instability and blebbing, ultimately leading to abnormal cytokinesis in the telophase stage.^21^, ^22^ Additionally, TEFT perturbs spindle microtubules and normal spindle assembly during mitosis, thereby preventing complete cytoplasmic separation.^10^

Apoptosis has traditionally been regarded as the sole form of controlled cell death, characterized by the disintegration of the nuclear membrane, cleavage of intracellular proteins, membrane blebbing, and the degradation of genomic DNA into nucleosomal structures.^23^ The induction of apoptotic cells by TEFT was found to be notably mediated by caspase‐3 activation and Poly (ADP‐ribose) Polymerase (PARP)‐1 cleavage, in a p53‐dependent manner.^24^, ^25^

Autophagy serves a dual function in the progression of tumors, as it promotes both tumor survival and growth by overcoming stressful conditions, while also suppressing tumor growth through the maintenance of cellular homeostasis at a basal level of autophagy.^26^ In consistency, the specific impact of autophagy in combinatorial therapy with TEFT remains unclear, as it is uncertain whether autophagy enhances or reduces the killing of GBM cells.^16^ It has been established that TEFT leads to mitotic arrest, which is associated with increased activation of autophagy.^27^ The induction of autophagic cell death by TEFT occurs through the miR‐29b‐Akt2 pathway, with downstream effects on the mammalian target of rapamycin (mTOR)/ribosomal protein S6 kinase (S6K)/eukaryotic translation initiation factor 4E binding protein 1 (4EBP1) axis.^25^ TEFT was found to enhance autophagic flux through the upregulation of proteotoxic stress response and the activation of AMP‐activated protein kinase (AMPK) and sequential unc‐51‐like autophagy‐activating kinase 1 (ULK1).^28^

### 2.2 TEFT inhibits DNA repair

Additionally, TEFT demonstrated inhibitory effects on DNA repair, as it suppressed the DNA damage response following exposure to radiotherapy,^29^ suggesting that a combination of radiotherapy and TEFT may be beneficial in controlling the progression of GBM. The breast cancer susceptibility gene 1 (BRCA1) gene, which plays a crucial role in DNA damage response, including repair of double‐strand DNA breaks and stalled fork repair. Knockdown of the BRCA1 gene resulted in an increase in R‐loops, DNA damage, and replication stress.^30^, ^31^, ^32^ The inhibition of the BRCA1 pathway was observed following exposure to TEFT.^33^ An increase in replication protein A (RPA), which serves as a marker for replication stress and protects single‐stranded DNA at stalled replication forks, was detected after TEFT exposure.^30^ These findings suggest that TEFT leads to an elevated level of DNA damage and a decrease in the capacity for repair through multiple pathways.^16^

### 2.3 TEFT inhibits cell proliferation and migration

TEFT suppressed GBM cell proliferation by reducing circMMD synthesis, thereby inhibiting the Wnt/β‐catenin pathway.^34^ Kirson et al. demonstrated that TEFT had the potential ability to inhibit the migration of tumor metastasis and activate antitumor immune response in peri‐tumoral location.^35^ Additionally, TEFT was found to impair the migration and invasion of GBM cells.^36^ Yoon et al. demonstrated that TEFT exerts inhibitory effects on cell migration and invasion by downregulating of phosphoinositide 3‐kinase (PI3K)/AKT/nuclear factor‐κB (NF‐ κB) signaling pathway.^37^

### 2.4 TEFT regulates immune response

GBM fostered an immunosuppressive environment characterized by dysfunction of T cells, inactivation of natural killer cells, elevated levels of regulatory T cells (Tregs), and myeloid lineage cells, including tumor‐associated macrophages (TAM), myeloid‐derived suppressor cells (MDSCs), and neutrophils.^38^, ^39^ In addition, immunosuppression is a key aspect of escaping immune recognition which was also partly induced by low immunogenicity, antigenic modulation, and immune‐privileged site.^40^ TAM played a vital role in immune escape in GBM through upregulating programmed death ligand 1(PD‐L1) expressed on GBM and activating programmed death 1(PD‐1) expressed on TAM.^40^, ^41^, ^42^ A range of chemokine chemotactic factors, such as alkB homolog 5, C‐C motif chemokine ligand 2/5 (CCL2/5), chitinase‐3‐like protein 1 (CHI3L1), C‐X3‐C motif chemokine ligand 1 (CX3CL1), and C‐X‐C motif chemokine ligand 8 (CXCL8) expressed and secreted by GBM cells, induce intratumoral immune suppression via promoting TAM infiltration and immunosuppressive polarization.^39^, ^43^ Combination of TEFT and anti‐PD‐1 therapy induced antitumor immune response,^44^ which may block the GBM immune evasion.

In the TEFT‐treated mouse model, there was a decrease in exhausted CD8+ T cells and an increase in the formation of memory T cells.^45^ Furthermore, patients who received TEFT treatment exhibited clonal expansion of T cells in their blood, indicating a robust tumor‐specific immune response.^45^ Positive T cell‐mediated responses were observed in TEFT‐treated tumor areas, as evidenced by CD45 activation and subsequent tumor necrosis factor (TNF)‐α production to induce cell death.^35^, ^46^ T‐lymphocyte counts have been identified as a prognostic indicator for treatment outcomes in the context of TEFT.^47^ Additionally, TEFT treatment has been shown to recruit dendritic cells (DCs) from the bone marrow, enhance the ability of bone marrow‐derived DCs to engulf cancer cells, and facilitate the maturation of DCs by upregulating MHC class II molecules, CD40, and CD80.^48^ Furthermore, TEFT has been found to elevate the levels of pro‐inflammatory cytokines, including interleukin (IL)‐1β, TNF‐α, and IL‐6, in macrophages through the regulation of the mitogen‐activated protein kinase (MAPK) and NF‐κB signaling pathway.^48^, ^49^ Moreover, TEFT‐treated macrophages have exhibited increased production of nitric oxide and reactive oxygen species (ROS), which have been shown to effectively eliminate tumor cells and pathogens.^49^

Due to the presence of the blood–brain barrier (BBB), the transport of activated immune cells from blood to the brain parenchyma was strictly restricted.^50^ TEFT increases the permeability of BBB,^51^, ^52^ which is an advantage factor for immune cells to access the brain parenchyma.

As mentioned above, TEFT has a powerful impact on promoting antitumor immunity to invert the immune‐suppressive environment. Furthermore, the promotion of immunogenic cell death (ICD) by TEFT emerged as a significant concern.^53^ ICD causes the liberation of specific molecules to activate immune response,^54^ which improves tumor immunogenicity.

TEFT induced ICD, characterized by the translocation calreticulin (CRT) to the cell surface, the release of the alarmin high‐mobility group box 1 (HMGB1), and the secretion of adenosine triphosphate (ATP).^44^ Voloshin et al. demonstrated that TEFT induced ICD via influencing the biological behavior of immune cells, such as the maturation of DCs in vitro and leukocyte recruitment *in vivo*.^44^ TEFT was found to activate the GMP‐AMP synthase (cGAS)/stimulator of interferon genes (STING) inflammasomes and absent in melanoma 2 (AIM2)/caspase‐1 inflammasomes, resulting in the production of pro‐inflammatory cytokines (PICs) and type 1 interferon (T1IFNs), which induced adaptive immunity against GBM.^55^

**FIGURE 1:**
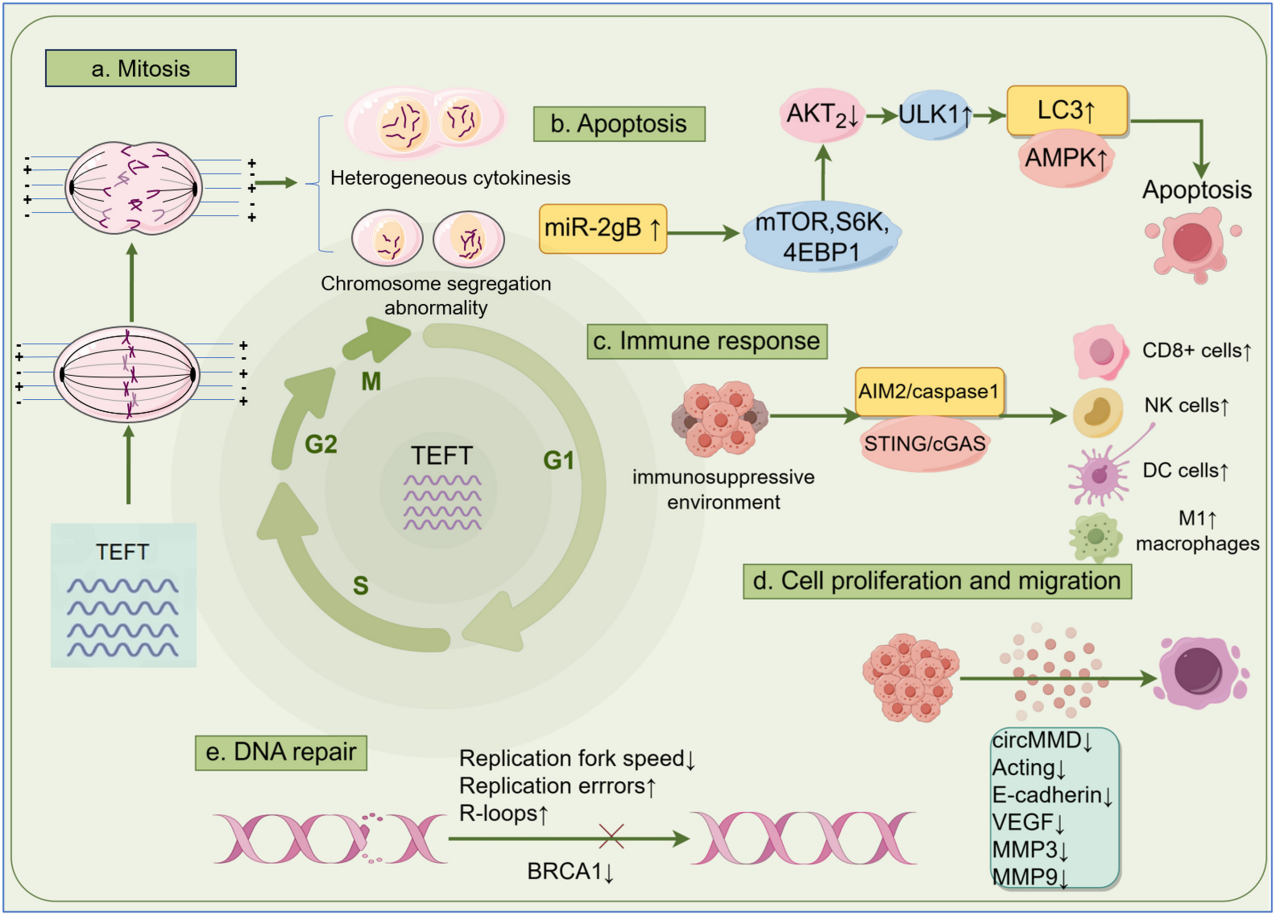
The mechanism of TEFT. TEFT inhibited GBM progression by disturbing mitosis, promoting apoptosis, triggering immune response, inhibiting DNA repair, and restraining cell proliferation and migration (By Figdraw).

The findings indicated that TEFT effectively inhibited the growth of GBM cells through a complex interplay of multiple factors, as depicted in Figure 1. While much research has focused on TEFT's role in mitotic arrest and cell death, further investigation is warranted to elucidate its mechanisms in stimulating immune activities.

## 3 EFFICACY OF TEFT

TEFT was employed to generate an alternating electric field using transducer arrays directly applied to the scalp of patients.^36^ The frequency range of TEFT, spanning from 10 kHz to 1 MHz, was carefully selected to prevent the stimulation of excitable tissues such as nerves and muscles.^36^, ^56^ It should be noted that high‐frequency fields exceeding 500 kHz were found to induce tissue heating via the vibration of charged and/or polar molecules.^16^, ^57^ In a study conducted by Kirson et al., it was demonstrated that TEFT within the frequency range of 100–300 kHz effectively inhibited the growth of GBM cells both in vitro and in vivo.^8^ Consequently, these intermediate‐frequency alternating electric fields (100–300 kHz) were deemed to be efficacious without causing any adverse tissue effects.

**FIGURE 2:**
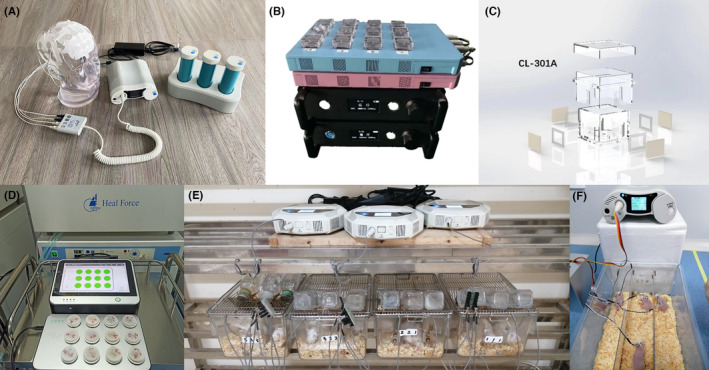
The current system of TEFT instrument. (A) The head model of TEFT instruments for clinical application (ASCLU‐350). (B) The first generation of TEFT instrument for cell culturing. (C) The quadrilateral petri dish for cells in TEFT instruments (CL‐301A). (D) The second generation of TEFT instruments for cell culturing (BES‐100). (E, F) The TEFT instruments for mouse.

The maximal inhibition of TEFT was found to be contingent upon the specific frequency of alternating electric fields, varying according to cell types.^8^ In the clinical therapy of GBM cells, a frequency of 200 kHz has been employed.^9^ Kseeler et al. proved that the frequency of 200 kHz had the maximum effect on four GBM cell lines (GaMG, U‐138MG, U‐343 MG, and U‐87 MG) proliferation among frequencies of 100, 200, 300, and 400 kHz.^58^ It should be noted that different patients with GBM displayed distinct characteristics, resulting in varying sensitive frequencies. Our research team has observed that the majority of cell lines exhibited sensitivity to a frequency of 200 kHz. However, the specific sensitive frequency varied for each cell line, and the therapeutic effect was enhanced by the random sequential sequence of TEFT. Furthermore, increasing the random sequential directions demonstrated improved efficacy in inhibiting tumor growth.^11^ Based on this result, Dr. Ling Chen's team developed a new type of TEFT equipment system named ASCLU‐300 which offers adjustable frequency and intensity along with random sequential direction.^59^ Dr. Ling Chen's team also upgraded the second generation of an instrument named ASCLU‐350 (Hunan An Tai Kang Cheng Biotechnology Co., Ltd.) (Figure 2), and we conducted a prospective, single‐center, single‐arm, exploratory study (NCT0441793).^60^, ^61^

5‐aminolevulinic acid hydrochloride was approved for use as an optical imaging agent in the context of neurosurgery for glioma resection to demarcate the relative boundary of malignant tissue and normal tissue.^62^ Exposure to TEFT resulted in heightened uptake of 5‐aminolevulinic acid, with this increase being directly proportional to the duration of exposure, owing to the augmented permeability of cellular membranes.^63^ 5‐aminolevulinic acid proved to be a reliable indicator for assessing the permeability of glioma cells, including GBM cells. Furthermore, TEFT significantly amplified both the quantity and size of cell membrane perforations, as observed through scanning electron microscopy.^63^ The efficacy of cooperative reinforcement between TEFT and chemotherapy might be implemented by TEFT improving the concentration of chemotherapeutic drugs within tumor cells. However, other studies have demonstrated that TEFT not only reduces the viability of multi‐drug resistant cells but also improves chemotherapy efficacy without impacting drug transport.^64^ These findings consistently indicate that TEFT increases the permeability of GBM cell membranes, thereby enhancing sensitivity to chemotherapy.

TEFT‐based combination therapies show promising potential for the treatment of GBM in the future. Here, we provide a comprehensive overview of preclinical research and clinical trials investigating the combination of TEFT with various treatment modalities, including chemotherapy, radiotherapy, concurrent chemoradiotherapy, targeted therapy, immunotherapy, small molecular inhibitors, tumor vaccine, skull remodeling surgery, and multiple‐treatment approaches (Table 1).

**TABLE 1:** Summary of clinical trials of TEFT.

Type of study	Authors or NCT number	Disease setting	Intervention	Outcomes	Enrollment	Status
Phase 2 clinical trial	NCT04671459	Recurrent glioblastoma	Combination product: TTFields and stereotactic radiosurgery	No results posted	40 patients	Recruiting
Phase 1 Clinical Trial	NCT03223103	Newly diagnosed glioblastoma	Drug: Poly‐ICLC Device: Tumor‐treating fields biological: peptides	No results posted	13 patients	Active, not recruiting
Phase 1 clinical trial	NCT02893137	Recurrent glioblastoma	Device: Optune Procedure: Craniectomy	No results posted	15 patients	Completed
Phase 1 clinical trial	NCT04474353	Newly diagnosed glioblastoma	Device: Optune Drug: GADOLINIUM Drug: Temozolomide Radiation: Stereotactic radiosurgery (SRS)	No results posted	12 patients	Recruiting
Phase 1 clinical trial	NCT03705351	Newly diagnosed glioblastoma	Device: tumor‐treating fields Drug: Temozolomide Radiation: Radiation therapy	Results information is not yet publicly available	7 patients	Active, not recruiting
Phase 1 clinical trial	NCT04397679	Newly diagnosed grade IV glioma	Radiation: 3‐dimensional conformal radiation therapy Radiation: Intensity‐Modulated radiation therapy (IMRT) Drug: Temozolomide Drug: Chloroquine Procedure: Tumor‐treating fields therapy (TTF)	No results posted	10 patients	Recruiting
Phase 1 clinical trial	NCT02903069	Newly diagnosed WHO grade IV malignant glioma	Drug: MRZ Drug: TMZ Radiation: RT Device: Optune	No results posted	48 patients	Completed
Phase 1 Clinical Trial	NCT03687034	Glioblastoma	Drug: Temozolomide Device: Optune	No results posted	21 patients	Not yet recruiting
Phase 1 clinical trial	Eilon D. Kirson	Recurrent glioblastoma	Device: NovoTTF‐100A (NovoCure Ltd., Haifa, Israel)	OS: 62.2 weeks^9^	10 patients	Completed
Phase 2 clinical trial	NCT03869242	Newly diagnosed GBM	Device: NovoTTF‐200A Drug: Temozolomide Radiation therapy	No results posted	60 patients	Recruiting
Phase 2 clinical trial	NCT03405792	Newly diagnosis glioblastoma	Drug: Temozolomide (TMZ) Device: Optune System Drug: Pembrolizumab	No results posted	31 patients	Active, not recruiting
Phase 2 clinical trial	NCT02743078	Recurrent glioblastoma	Drug: Bevacizumab Device: TTFields therapy	Maximum follow‐up: 21.8 months. Skin and subcutaneous tissue disorders (Rash maculo‐papular) were reported in three (100%) patients	3 patients	Terminated (Treatment now available commercially)
Phase 2 clinical trial	NCT04221503	Recurrent glioblastoma	Drug: Niraparib Device: Optune Procedure: Planned surgical resection	No results posted	30 patients	Active, not recruiting
Phase 2 clinical trial	NCT04469075	Glioblastoma	Drug: Clindamycin phosphate Drug: Triamcinolone acetonide	No results posted	58 patients	Recruiting
Phase 2 clinical trial	NCT04223999	Recurrent glioblastoma	Procedure: Skull remodeling surgery Other: Control	No results posted	70 patients	Recruiting
Phase 2 clinical trial	NCT01894061	Recurrent glioblastoma	Biological: Bevacizumab Device: NovoTTF‐l00A Other: Quality of life assessment	No results posted	25 patients	Completed
Phase 2 clinical trial	NCT02343549	Newly diagnosed unresectable glioblastoma	Device: NovoTTF100A Drug: Bevacizumab Drug: Temozolomide	PFS: 7.9 months	13 patients	Terminated (closed to accrual due to low accrual)
Phase 2 clinical trial	NCT03430791	Recurrent glioblastoma	Drug: Nivolumab 240 mg IV Drug: Nivolumab 3 mg/kg Drug: Ipilimumab 1 mg/kg Device: NovoTTF200A (Optune)	PFS: 62.5 days	5 patients	Terminated (Study Investigator/Sponsor decided to end enrollment earlier)
Phase 2 clinical trial	NCT02663271	Recurrent glioblastoma	Drug: Bevacizumab Device: Optune Other: Brain MRI Other: Quality of LIFE QUESTIONNAIRES	OS: 7.4 months	10 patients	Terminated (stagnant enrollment)
Phase 3 clinical trial	NCT00916409	Newly diagnosed GBM	Device: NovoTTF‐100A device Drug: Temozolomide	Median PFS: 6.7 months^14^	695 patients	Completed
Clinical trial (phase is not available)	NCT04218019	Newly diagnosed glioblastoma in patients ≥70 Years	Device: TTFields Short‐course radiation	No results posted	68 patients	Suspended (Organizational reasons)
Clinical trial (Phase is not available)	NCT03780569	Newly Diagnosed GBM	Device: NovoTTF‐200A Radiation: Radiotherapy Drug: Temozolomide	PFS: 6.7 months; OS: 20.9 months^91^	10 patients	Active, not recruiting
Clinical trial (Phase is not available)	NCT01925573	Recurrent glioblastoma	Device: Optune (NOVOTTF‐100A) Drug: Bevacizumab Hypofractionated stereotactic irradiation	<50% rate of Grade 2–3 scalp dermatitis in 6 months	7 patients	Terminated (Poor accrual)
Clinical trial (phase is not available)	NCT04471844	Newly diagnosed glioblastoma	Device: Optune® Drug: Temozolomide Radiation therapy	No results posted	950 patients	Recruiting

*Note*: 36 studies found for TTFields|glioblastoma in ClinicalTrial.gov.

Abbreviations: OS, overall survival; PFS, progression‐free survival.

Stupp et al. reported that compared to active chemotherapy, the median survival of TEFT application alone only prolonged 0.6 months, and the difference was not significant (*p* = 0.27).^7^ The result meant the curative effect of TEFT was equivalent to chemotherapy, and the combination of those two entirely different therapies might be quite effective. A phase 3 (EF‐11) randomized clinical trial in 695 newly diagnosed GBM patients concluded that combination therapy with TEFT and chemotherapy was more effective than chemotherapy alone (median OS of 20.9 months vs. 16.0 months).^14^ A second phase 3 (EF‐14) randomized clinical trial for newly diagnosed GBM revealed that the combination of TEFT (≥ 18 h/d) and TMZ maintenance therapy significantly prolonged the OS with 4.9 months compared to TMZ alone group (20.9 months vs. 16 months).^14^ The median time of this trial from diagnosis to randomization was 3.8 months in the combination of TEFT plus TMZ group and 3.7 months for TMZ alone group. Accordingly, the median OS for the patients receiving TEFT plus TMZ was 24.7 months from the diagnosis.^65^

Kim et al. found that radiotherapy enhanced cellular response as TEFT was administrated prior to radiotherapy.^24^ In contrast, delaying TEFT application after radiotherapy also increased treatment efficacy, and the combination of TEFT and radiotherapy showed no increase in skin toxicities.^29^ Furthermore, the combination of spindle assembly checkpoint (SAC) inhibitor MPS1‐IN‐3 (IN‐3) and TEFT resulted in a stronger impact on GBM cell lines with an increased apoptotic rate compared to TEFT or IN‐3 treatment alone.^58^ Additionally, the concurrent application of TEFT and anti‐PD‐1 therapy was found to be safe without causing pathological changes in normal lungs and decreased tumor volume, albeit without statistical significance when compared to monotherapy of TEFT or anti‐PD‐1. This was accompanied by an increase in macrophages, DCs infiltration, and interferon (IFN)‐γ production in vivo.^44^ Although the experiment was conducted on mice with lung carcinoma and the inhibitory effect of combination therapy on tumor growth did not reach statistical significance, the findings demonstrated the safety of combination therapy and indicated a potential for inhibiting tumor growth. These results hold valuable implications for GBM research.

## 4 ADVANTAGES OF TEFT

Chemotherapy is commonly used as an adjunctive treatment for post‐resection GBM. However, the efficacy of chemotherapy is hindered by the limited permeability of BBB. Although long‐term or high‐dose chemotherapy has shown effectiveness in killing GBM cells and preventing recurrence, it also induces toxic effects and drug resistance.^66^ In certain cases, the development of systemic toxicity, such as myelosuppression, necessitates discontinuation of chemotherapy. Compared to chemotherapy, radiotherapy exhibits lower levels of systemic toxicity and serves as a vital physical treatment modality for eliminating residual microscopic lesions after surgical resection and preventing the recurrence of GBM. Patients undergoing cerebral radiotherapy frequently experience a complication known as radiation‐induced brain injury, which contributes to the clinical presentation of increased intracranial pressure.^67^ Besides, radiation may also induce systemic toxicity, such as lymphopenia, thrombocytopenia, alopecia, fatigue, cognitive impairment, and memory loss.^67^ Though immunotherapy, including immune checkpoint blockade, oncolytic therapy, and vaccine therapy, has made significant progress in some cancers, the research on immunotherapy for GBMs is still being conducted.^68^ The targeted agents derived from the antitumoral immune response also induce inflammatory and anti‐immune side effects.^69^

TEFT offers a non‐invasion and portable approach to prolong survival time with fewer side effects. TEFT instruments are accessible to be carried, which means patients can receive tumor‐treating fields at their convenience. In contrast to both chemotherapy and radiotherapy could induce resistant GBM cells through enhancing DNA damage response, the existence of GBM stem cells, and remolding the tumor microenvironment,^70^ TEFT has few side effects and rare resistance. Our research team found that long‐term TEFT does not adversely affect vital organs and tissues, such as kidney, liver, and blood.^59^ Moreover, clinical trials revealed that the addition of TEFT to TMZ therapy did not exhibit a significant correlation with the occurrence or intensity of systemic adverse events.^14^ However, it should be noted that the utilization of TEFT devices may result in a higher occurrence of adverse events specifically related to dermal toxicity beneath the transducer arrays (Table 2).^14^ Consequently, it can be concluded that TEFT represents a viable, secure, and user‐friendly therapeutic approach for patients diagnosed with GBM.

**TABLE 2:** Comparison of adverse effects between TEFT and other conventional therapies.

	TEFT^7^	Chemotherapy^92^	Radiotherapy^93^, ^94^	Immunotherapy^95^, ^96^, ^97^, ^98^, ^99^, ^100^, ^101^, ^102^	Targeted therapy^103^, ^104^, ^105^, ^106^, ^107^
Skin injury	✓	✓		✓	✓
Gastrointestinal disorder		✓		✓	✓
Fatigue		✓		✓	✓
Headache		✓	✓	✓	✓
Blood cell disorder		✓		✓	✓
Elevated intracranial pressure			✓		
Dizziness			✓	✓	
Injection‐site reaction				✓	
Epilepsy			✓	✓	
Myalgia (muscle pain) or arthralgia				✓	✓
Cardiac disorder				✓	
Fever				✓	✓
Infusion reaction				✓	✓
Peripheral motor neuropathy				✓	
Migraine				✓	
Meningitis with hydrocephalus				✓	
Respiratory system disorder				✓	✓
Hepatic insufficiency				✓	✓
Electrolyte disturbance					✓
Leukoencephalopathy			✓		
Neurologic deficits			✓	✓	

## 5 THE PROSPECTIVELY INNOVATIVE APPLICATION OF TEFT AND DISCUSSION

**FIGURE 3:**
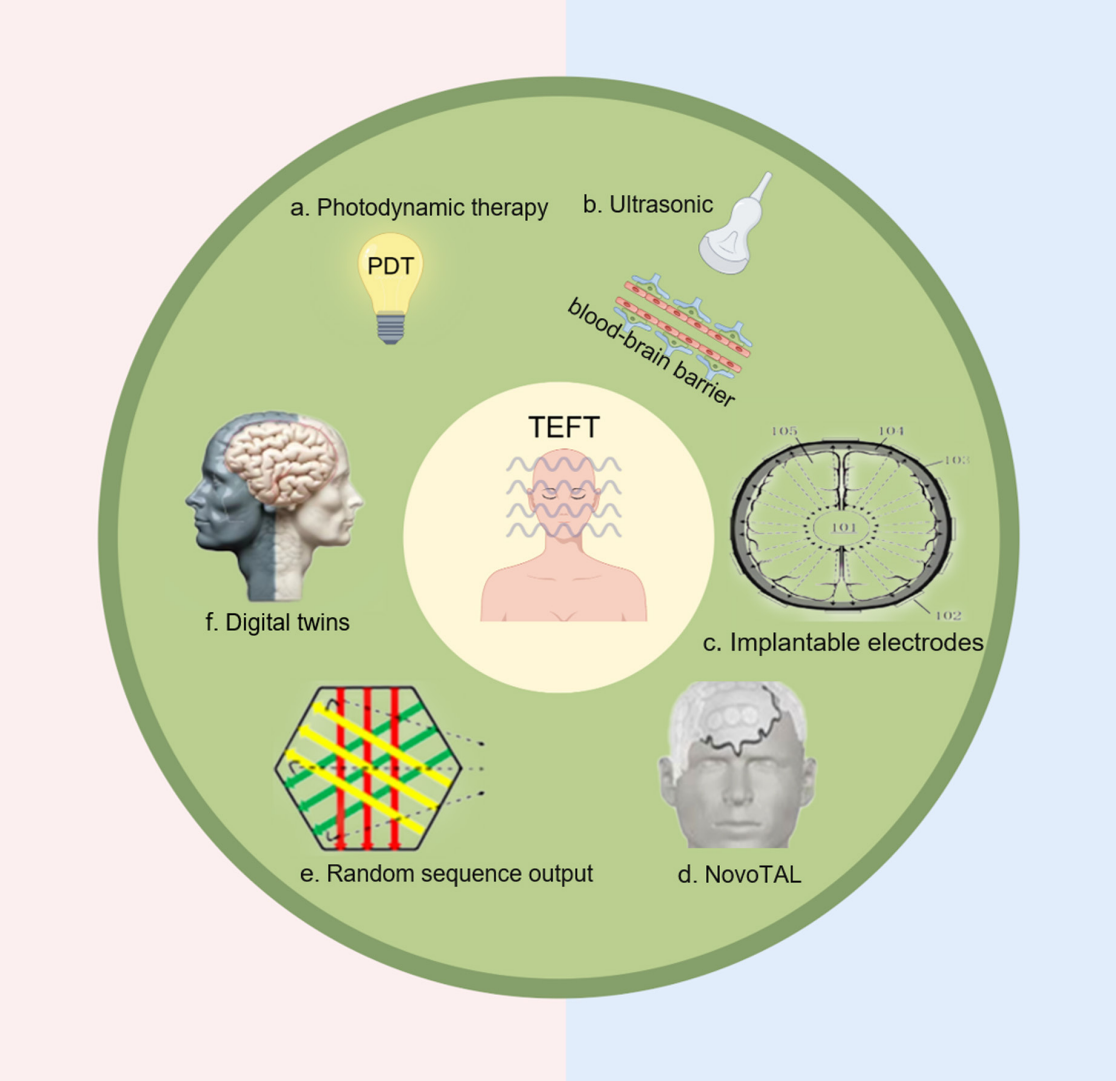
The prospect of TEFT plus advanced therapy (By Figdraw).

The development of TEFT as an innovative therapeutic approach for GBM has prompted the exploration of its full potential through the utilization of advanced iatrotechnique for tumor treatment. To provide a comprehensive overview, we have compiled prospective patents related to TEFT (Table 3) and have also presented a visual representation in the form of a blueprint (Figure 3).

**TABLE 3:** Patents of emerging technologies for TEFT.

	Title	Date of filing	Application number	Notification number or publication patent number	Publication patent date
1	Reducing motility of cancer cells using tumor‐treating fields (TTFields)^81^	2017.04.04	EP19219639	EP3693054B1	2023.06.28
2	Arrays for delivering tumor‐treating fields (TTFields) with selectively addressable sub‐elements^108^	2019.11.18	US16686918	US20200155835A1	2020.05.21
4	Arrays for delivering tumor‐treating fields (TTFields) with individually accessible electrode elements and temperature sensors^86^	2020.12.21	PCT/IB2020/062309	WO/2021/137094	2021.07.08
5	Temperature measurement in arrays for delivering TTFields^87^	2017.08.11	PCT/IB2017/054922	WO/2018/033842	2018.02.22
6	TTFields treatment with optimization of electrode positions on the head based on MRI‐based conductivity measurements^76^	2016.10.27	US201615336660	US2017120041A1	2017.05.04
7	Determining a frequency for TTFields treatment based on an electrical characteristic of target cancer cells^109^	2020.02.25	US16800737	US20200269042A1	2020.08.27
8	Evaluating the quality of segmentation of an Image into different types of tissue for planning treatment using tumor‐treating fields (TTFields)	2020.01.07	US16736604	US20200219261A1	2020.07.09
9	Optimizing treatment using TTFields by changing the frequency during long‐term tumor treatment^82^	2017.04.21	US201715493309	US2017215939A1	2018.08.03
10	Determining a frequency for TTFields treatment based on an electrical characteristic of targeted cancer cells^83^	2020.02.25	US16800737	US20200269042A1	2020.08.27
11	Treating cancer using electromagnetic fields in combination with photodynamic therapy^72^	2013.03.07	US201313788154	US9023090B2	2015.05.05
12	Using power loss density and related measures to quantify the dose of tumor‐treating fields (TTFields)^77^	2019.07.18	US16515311	US20200023179A1	2020.01.23
13	Delivering tumor‐treating fields (TTFields) using implantable transduce arrays^110^	2020.02.26	US16801972	US20200269043A1	2020.08.27

Photodynamic therapy (PDT) involves the utilization of a photosensitizer that is exposed to appropriate illumination light and energy, resulting in the generation of cytotoxic reactive oxygen species (ROS) and subsequent cell death.^71^ The photosensitizers exhibit a preference for localizing in tumors rather than normal cells, thereby enabling photodynamic therapy to specifically target and eliminate tumor cells. By disturbing the proliferation of dividing cells, the antitumor effect induced by TEFT could be reinforced with combination of photodynamic therapy. Suitable light sources for PDT, such as light‐emitting diodes (LEDs), are activated by applying an alternating current field with specific orientation and intensity. By adjusting the field orientation or intensity below the LED turn‐on threshold, TEFT can be performed without activating PDT.^72^

The BBB can be effectively opened by focused ultrasound, excluding vessels with a diameter exceeding 30 μm.^73^ In a phase 1 clinical trial involving adults with recurrent GBM, the BBB was successfully opened using a skull‐implantable ultrasound device combined with intravenous microbubbles (LIPU‐MB).^74^ The potential of enhancing the reversible opening of the BBB and increasing drug concentration could be further amplified by combining the ultrasound device with TEFT, thereby potentially augmenting the efficacy of chemotherapy drugs.

In conjunction with the integration of scalp electrodes, the universal implantable electrodes generate an electrical field with multiple stereoscopic orientations. This configuration allows for reduced distance to the lesion, independent of the anatomical structure of the scalp and skull, thereby requiring lower voltage and enhancing safety and effectiveness. It is prospective that integrate the implantable electrodes with implantable ultrasound device to form a unified device.

Immunotherapy, encompassing vaccines and adoptive immune cell transfer therapy, represents emerging treatment modalities for GBM patients.^75^ The implantation of an Ommaya reservoir serves as a pivotal approach for immunotherapy, facilitating the delivery of chimeric antigen receptor (CAR) T cells into the cerebrospinal fluid circulation. By integrating the comprehensive design of the Ommaya reservoir and TEFT implantable electrodes with an ultrasound device, a multifaceted therapeutic approach can be achieved, offering enhanced device functionality and mitigating patient distress through a singular surgical intervention.

As TEFT inhibited tumor growth via electric fields, the position of transducer array is vital to optimize the TEFT efficacy. To achieve this, a 3D map of electrical conductivity, resistivity, and power loss density can be generated using magnetic resonance imaging (MRI),^76^, ^77^ taking into account the anatomical volume of the target tissue. The utilization of MRI measurements in conjunction with the NovoTAL System (Novocure Ltd.) could adjust the maximal electric field intensity at the tumor site.^78^ Additionally, a theory of evaluating the quality of segmentation of MRI was developed, and it provided the best segmentation to determine transducer arrays layouts.^79^ Based on these techniques, adjusting the position of electrodes would elevate the efficacy of TEFT.

Single‐cell heterogeneity was inferred because of variable transcription and distinct GBM subtypes in the same tumor.^80^ Notably, the optimal frequencies for GBM varied among different patients and cell types.^8^, ^11^ The use of a fixed frequency in TEFT only inhibited specific cell types, while exhibiting a weaker inhibitory effect on other heterogeneous cells. Conversely, employing a random frequency in TEFT enhanced the therapeutic effect on GBM,^11^ suggesting that the application of a random frequency in TEFT may effectively target heterogeneous cells with varying optimal frequencies. The theory was transferred to a utility device reducing the motility of GBM cells with changing frequency and amplitude every one second.^81^ Besides, changing the direction of TEFT also enhanced the therapeutic efficacy through promoting cell apoptosis and CD8+ T cell infiltration.^11^ The selection of the frequency of the alternating electric field can be determined based on the size of the cells through biopsy or inverse electric impedance tomography^82^ and electrical characteristics of GBM cells obtained from the patient.^83^ The implementation of individualized treatment frequencies, the adjustment of switching frequency according to multiple optimal frequencies, and the incorporation of multidirectional mode are expected to significantly enhance the effectiveness of TEFT.

Though there were multiple technologies to adjust the position of electrodes to exert the maximal field density on GBM under the same voltage, the prediction of the TEFT treatment efficacy for individual patient was still rarely developed. Digital twins were the virtual counterparts of the entity measures, which witnessed the status of a physical object much earlier for further optimization.^84^, ^85^ The application of digital twins could simulate the TEFT and provide the prediction of TEFT efficacy. According to the predictive data, we could adjust the parameters of TEFT equipment, such as the position of electrodes, voltage, and current. As the electrodes of TEFT generating heat during producing electric fields, the sensors monitoring the temperature of electrodes transmitted the data to a central hub and switched off the overheating electrodes.^86^, ^87^ Digital twin could substitute the role of the central hub to calculate and predict the temperature of electrodes. The efficacy of TEFT was dependent on the compliance of patients^88^ and the duration of effective field intensity. As the TEFT equipment was connected to the network and transferred data to digital twin appliance, digital twins could also monitor the use duration of TEFT, especially elevating the duration of effective field intensity. This technology could improve the efficacy of TEFT for supervising all stages of TEFT usage.

In this study, we mainly summarized the innovation and prospective application of TEFT. Previous studies had shown that TEFT inhibited GBM growth in vitro and in vivo,^9^, ^11^ and as discussed above, the mechanisms of TEFT were not fully understood. The researches on mechanisms of TEFT acting on crosstalk between GBM tumor microenvironment would be a hotspot of research field. It is a critical point that develops a TEFT device for cell co‐culture and monitoring culture medium component. Neural stem cells (NSCs), mainly existing in the subventricular zone (SVZ), are associated with the origin and recurrence of GBM.^89^, ^90^ It is worth exploring that the antitumoral effect of TEFT on NSCs located in SVZ.

## AUTHOR CONTRIBUTIONS

Conceived and designed the review: JLL and LC. Wrote and revised the paper: JXL, YYL, and JYC. All authors contributed to the article and approved the submitted version.

## FUNDING INFORMATION

This project has been supported by National Natural Science Foundation of China (Grant/Award Nos. 82303586, 82373220, 82172680).

## CONFLICT OF INTEREST STATEMENT

The authors declare that the research was conducted in the absence of any commercial or financial relationships that could be construed as a potential conflict of interest.

Ling Chen is an Editorial Board member of CNS Neuroscience and Therapeutics and a co‐author of this article. To minimize bias, they were excluded from all editorial decision‐making related to the acceptance of this article for publication.

## Data Availability

Data sharing not applicable to this article as no datasets were generated or analysed during the current study.
